# Supplementary figures and images for: Tad pilus-mediated twitching motility is essential for DNA uptake and survival of Liberibacters
Source: PLoS One. 2021 Oct 13;16(10):e0258583. doi: 10.1371/journal.pone.0258583 (PMC8513845; doi:10.1371/journal.pone.0258583)

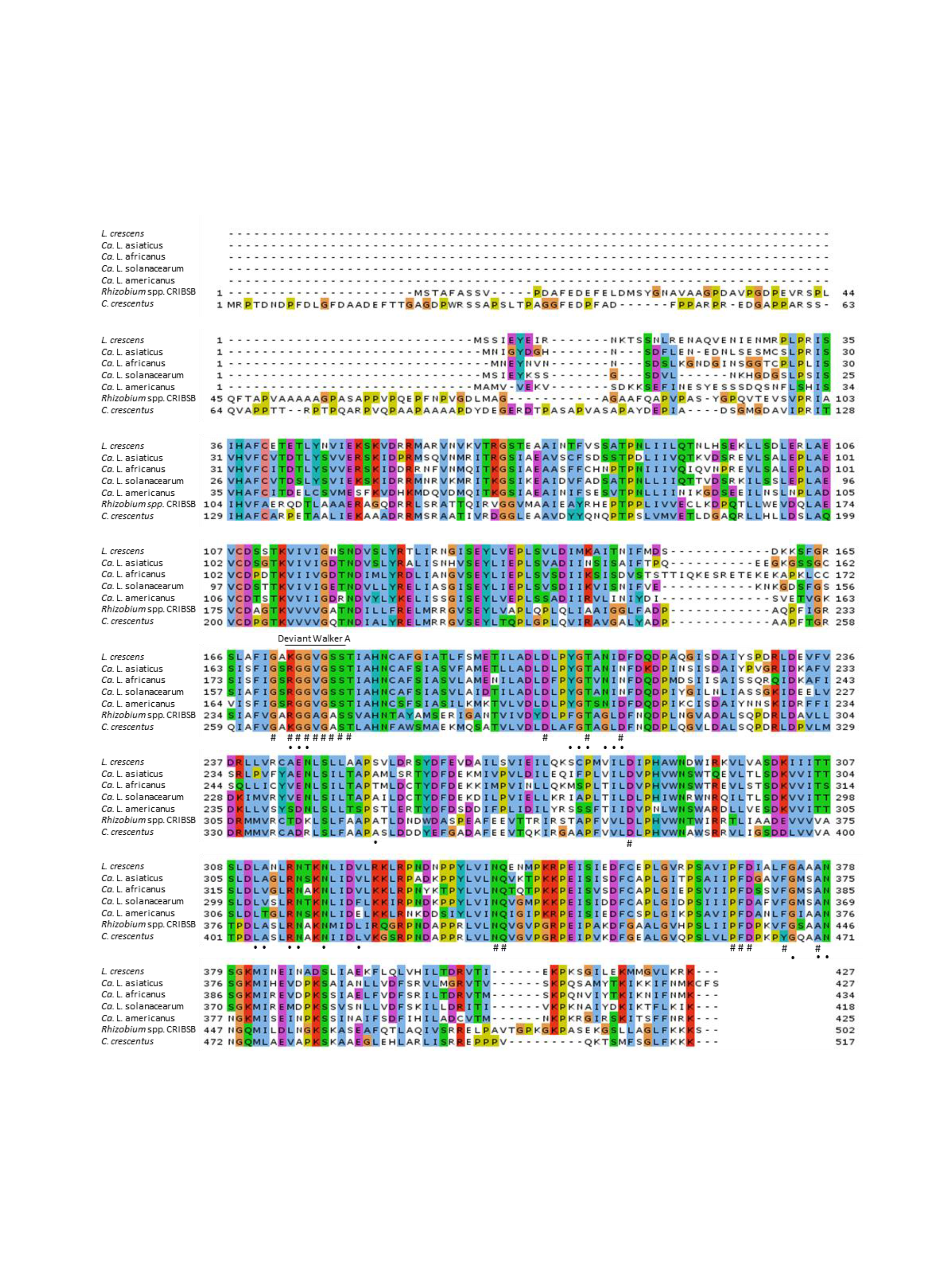

Supplement: S1 Fig — Sequence alignment of CpaE encoded by L. crescens BT‐1 (WP_015273699.1), ‘Ca. L. asiaticus’ (WP_015452437.1), ‘Ca. L. africanus’ (WP_047264074.1), ‘Ca. L. solanacearum’ (WP_103846917.1), ‘Ca. L. americanus’ (WP_144079396.1), Rhizobium spp. CRIBSB (WP_166603675.1) and Caulobacter crescentus (YP_002518411.1). Deviant ATP-binding Walker A motif is marked. Amino acids participating in ATP binding and ATPase activity are denoted by hashtag (#) and residues involved in dimer interface are denoted by dots (·). The ‘signature’ K173 residue in Lcr CpaE is expected to mediate homodimerization by binding to the phosphates of ATP engaged by the other subunit. The deviant Walker A motif of all pathogenic ‘Ca. Liberibacter’ spp. diverges further, where the ‘signature’ K residue is replaced by similar, positively charged and small sized R or H residues. (TIF) [file pone.0258583.s001.tif]

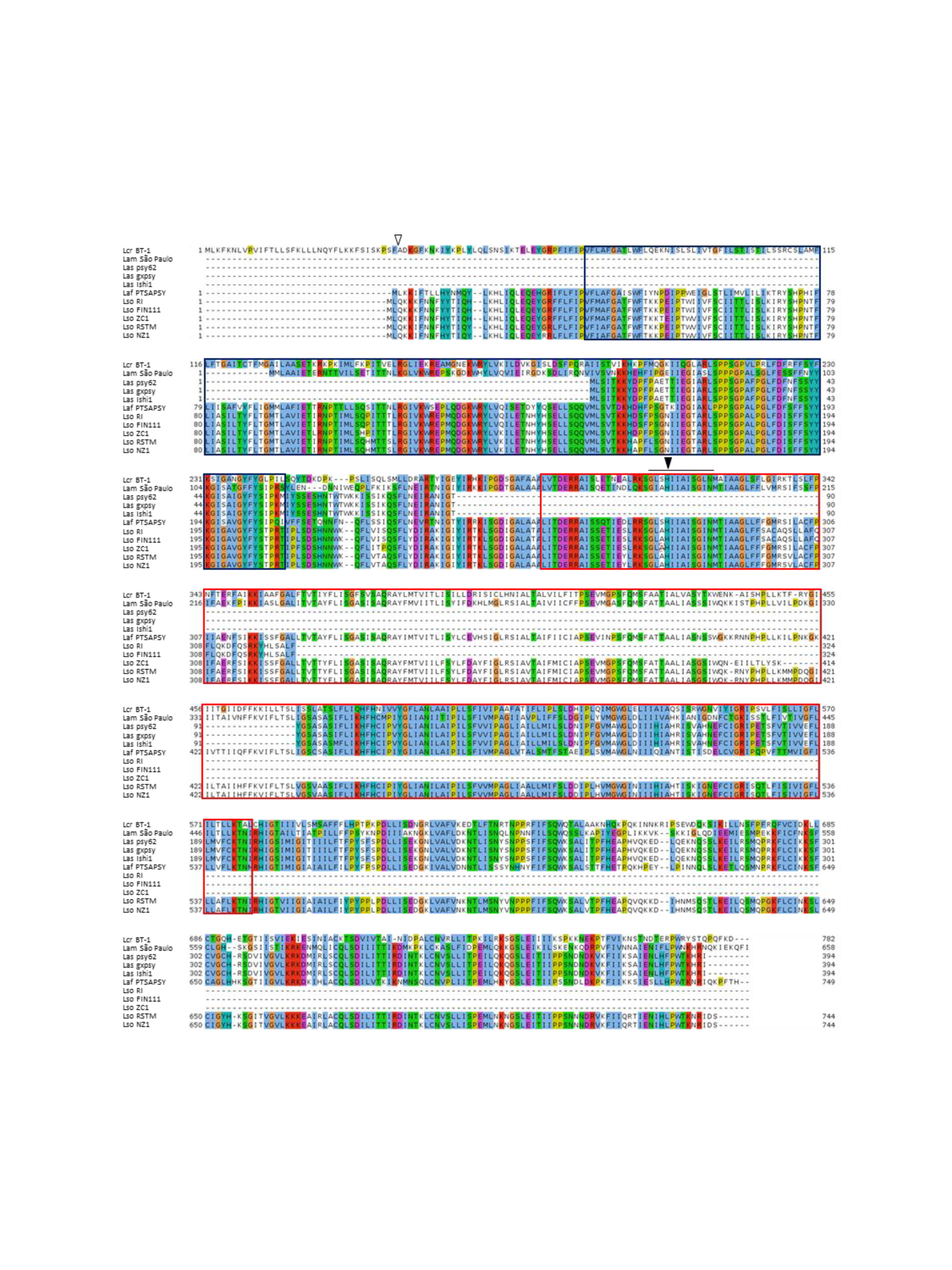

Supplement: S2 Fig — The following sequences were used for alignment: Lcr strain BT1 (WP_015273513.1), ‘Ca. L. asiaticus’ (Las) strains psy62 (WP_012778576.1), Ishi-1 (WP_045490146.1) and gxpsy (WP_012778576.1-like), ‘Ca. L. solanacearum’ (Lso) strains ZC1 (WP_080550987.1), RI (ONI59307.1), FIN111 (WP_076969511.1), RSTM (WP_055347849.1) and NZ1 (KJZ82350.1), ‘Ca. L. africanus’ (Laf) strain PTSAPSY (WP_052775004.1) and ‘Ca. L. americanus’ (Lam) strain Sao Paulo (AHA27693.1). The N-terminal domain of unknown function DUF4131 followed by the universal transmembrane competence domain are represented by black and red boxes, respectively. The metal-binding motif is underlined. The invariant H314 and the signal peptide cleavage sites (in Lcr) are denoted by open (△) and filled (▲) triangles, respectively. (TIF) [file pone.0258583.s002.tif]
