# Supplementary material for: Tad pilus-mediated twitching motility is essential for DNA uptake and survival of Liberibacters
Source: PLoS One. 2021 Oct 13;16(10):e0258583. doi: 10.1371/journal.pone.0258583 (PMC8513845; doi:10.1371/journal.pone.0258583)
Supplement: Attachment — Submitted filename: Response to Reviewers v2.docx [file pone.0258583.s003.docx]

PONE-D-21-23751

Tad pilus-mediated twitching motility is essential for DNA uptake and survival of Liberibacters.

PLOS ONE

Dear Dr. Gabriel,

Thank you for submitting your manuscript to PLOS ONE. After careful consideration, we feel that it has merit but does not fully meet PLOS ONE’s publication criteria as it currently stands. Therefore, we invite you to submit a revised version of the manuscript that addresses the points raised during the review process.

==============================

The MS is quite lengthy and needs shortening to improve the readability. Kindly refer to referee's suggestions and address carefully. Discussion and Introduction need to be shortened, and figures 5-6 should go to supplementals. Proposed Model must move to back of results to maintain the flow.

==============================

***OK. Manuscript now shortened in both discussion and introduction; Figs 5-6 moved to supplementals. Proposed model moved to end of results.***

Journal Requirements:

When submitting your revision, we need you to address these additional requirements.

2. In your Data Availability statement, you have not specified where the minimal data set underlying the results described in your manuscript can be found. PLOS defines a study's minimal data set as the underlying data used to reach the conclusions drawn in the manuscript and any additional data required to replicate the reported study findings in their entirety.

***Now clarified.***

3. PLOS ONE now requires that authors provide the original uncropped and unadjusted images underlying all blot or gel results reported in a submission’s figures or Supporting Information files. This policy and the journal’s other requirements for blot/gel reporting and figure preparation are described in detail at

<https://journals.plos.org/plosone/s/figures#loc-blot-and-gel-reporting-requirements><<https://urldefense.proofpoint.com/v2/url?u=https-3A__journals.plos.org_plosone_s_figures-23loc-2Dblot-2Dand-2Dgel-2Dreporting-2Drequirements&d=DwQGaQ&c=sJ6xIWYx-zLMB3EPkvcnVg&r=6Mm2ViKBl28xlMHnOORoBQ&m=zCC-_WY6MTr1xMJ1sgGNCWvXUDo5cwn2sj1JgzQyiHg&s=C6_BlGv_yeX_Dd4TLgAeIVqgVM1WxQHIYK_fHMUypDw&e=>> and

<https://journals.plos.org/plosone/s/figures#loc-preparing-figures-from-image-files><<https://urldefense.proofpoint.com/v2/url?u=https-3A__journals.plos.org_plosone_s_figures-23loc-2Dpreparing-2Dfigures-2Dfrom-2Dimage-2Dfiles&d=DwQGaQ&c=sJ6xIWYx-zLMB3EPkvcnVg&r=6Mm2ViKBl28xlMHnOORoBQ&m=zCC-_WY6MTr1xMJ1sgGNCWvXUDo5cwn2sj1JgzQyiHg&s=MEPufFjs_9ysUhW_hpdU3D80puYHXALLG-qG2rShY-o&e=>>. When you submit your revised manuscript, please ensure that yourfigures adhere fully to these guidelines and provide the original underlying images for all blot or gel data reported in your submission. See the following link for instructions on providing the original image data:

***We have no blot or gel images in our Figures.***

4. We note that you have included the phrase “data not shown” in your manuscript. Unfortunately, this does not meet our data sharing requirements. PLOS does not permit references to inaccessible data. We require that authors provide all relevant data within the paper, Supporting Information files, or in an acceptable, public repository. Please add a citation to support this phrase or upload the data that corresponds with these findings to a stable repository (such as Figshare or Dryad) and provide and URLs, DOIs, or accession numbers that may be used to access these data. Or, if the data are not a core part of the research being presented in your study, we ask that you remove the phrase that refers to these data.

5. Please upload a new copy of Figure 4, 5 and 6 as the detail is not clear. Please follow the link for more information:

***New Figures provided.***

Additional Editor Comments:

The MS is lengthy and needs shortening to improve the readability. Discussion and Introduction need to be shortened, and figures 5-6 should go to supplementals. Model can be moved to back of results to maintain the flow.

***OK. Manuscript now shortened in both discussion and introduction; Figs 5-6 moved to supplementals. Proposed model moved to end of results.***

Reviewers' comments:

Reviewer's Responses to Questions

Comments to the Author

1. Is the manuscript technically sound, and do the data support the conclusions? The manuscript must describe a technically sound piece of scientific research with data that supports the conclusions. Experiments must have been conducted rigorously, with appropriate controls, replication, and sample sizes. The conclusions must be drawn appropriately based on the data presented.

Reviewer #1: Yes

2. Has the statistical analysis been performed appropriately and rigorously?

Reviewer #1: Yes

3. Have the authors made all data underlying the findings in their manuscript fully

available?

Reviewer #1: Yes

4. Is the manuscript presented in an intelligible fashion and written in standard English?

Reviewer #1: Yes

5. Review Comments to the Author

Reviewer #1: The manuscript ‘Tad pilus-mediated twitching motility is essential for DNA uptake and survival of Liberibacters’ by Cai et al. describes that the Liberibacter Tad pili are essential for twitching motility and DNA uptake for dsDNA in Liberibacter crescens, which is used as a surrogate for unculturable plant pathogenic Liberibacters. Liberibacter are important plant pathogens. The manuscript is exhaustive, and authors have done a good job by providing the ultrastructural details of both- flagella as well as Tad pilus, and demonstrated the tad-pilus mediated uptake of extracellular DNA in L. crescens. This is important as Liberibacters cannot synthesise nucleotides and depend on DNA uptake for their survival.

The manuscript is based on a robust hypothesis, and the experimental evidence provided is adequate and sufficient to demonstrate that flagella and Tad pilus are present, are involved in swimming and twitching motility, respectively, and that the Tad pilus is involved in DNA uptake. The results from the mutation and complementation experiments supports the conclusions. The bioinformatic analysis is adequate and supports the conclusions.

I have following suggestion to improve the manuscript.

Introduction needs to be shortened.

***Now shortened.***

The results, especially the model and the bioinformatics results (lines 310 to 335, lines 347-355) have become more of discussion than results. I suggest that the model should be moved to the end of results, and all the discussion presented in the bioinformatics results should be moved to discussion section.

***Model now moved to end of results, and bioinformatics results appropriately shortened.***

The model (Figure 3) should be presented at the end of results after the bioinformatic and mutational analysis to preserve the flow of the manuscript.

***Done.***

Figures 5 and 6 may be moved to supplemental information.

***Done.***

The discussion may be shortened a bit and organised into subheadings for better readability.

***Done.***

________________________________
